# PATHWAYS TO OR WITHIN SOCIAL EXCLUSION? AN INTERSECTIONAL LIFE COURSE PERSPECTIVE ON OLDER ROMANIAN ROMA

**DOI:** 10.1093/geroni/igad104.0225

**Published:** 2023-12-21

**Authors:** Laura Tufă, Iuliana Precupetu, Ionut Anghel, Marja Aartsen

**Affiliations:** University of Bucharest, Bucharest, Bucuresti, Romania; Research Institute of the University of Bucharest, Bucharest, Bucuresti, Romania; Research Institute for Quality of Life, Romanian Academy, Bucharest, Bucuresti, Romania; OsloMet Oslo Metropolitan University, Oslo, Akershus, Norway

## Abstract

Social exclusion is considered a dynamic process as people can move in and out of exclusion while also experiencing different types of exclusion over their life course. In this paper we set out to understand pathways to social exclusion of older Romanian Roma, a particular population that received little attention in the literature. We aim to understand the lived experiences of social exclusion of Roma with the use of life histories and events calendars and we employ an intersectional life course perspective by looking at the nexus of structural, individual, and relational factors that impacted the pathways of older Roma across their life course. The intersection between ethnicity, gender, education, work, residence/segregation and income is employed in order to understand how disadvantage unfolded across the life course and into late life. The data come from 15 biographical interviews with older Roma living in rural and urban Romania, collected in 2021. Results illustrate how specific structural processes like sedentarization of nomad communities, the fall of the communist regime in 1989 and the retrocession of gold unevenly affected Roma across the intersectional characteristics and transformed into turning points in their lives, while interposing to a little extent with their pathways to social exclusion. De-institutionalized life courses and fast transitions had an important bearing on the ways disadvantage unfolded for older Roma living in segregated communities. Finally, super-linked lives at family and community level, when deprived of outside support, contributed to deep exclusion in later life.

